# Long-term effects of prenatal infection on the human brain: a prospective multimodal neuroimaging study

**DOI:** 10.1038/s41398-023-02597-x

**Published:** 2023-10-03

**Authors:** Anna Suleri, Charlotte Cecil, Anna-Sophie Rommel, Manon Hillegers, Tonya White, Lot D. de Witte, Ryan L. Muetzel, Veerle Bergink

**Affiliations:** 1 Department of Child and Adolescent Psychiatry/Psychology, Erasmus MC University Medical Center, Rotterdam, The Netherlands; 2 The Generation R Study Group, Erasmus MC University Medical Center, Rotterdam, the Netherlands; 3 Department of Psychiatry, Icahn School of Medicine at Mount Sinai, New York, USA; 4 Section on Social and Cognitive Developmental Neuroscience, National Institute of Mental Health, Bethesda, MD USA; 5 Department of Radiology and Nuclear Medicine, Erasmus MC University Medical Center, Rotterdam, The Netherlands; 6 Department of Psychiatry, Erasmus MC University Medical Center, Rotterdam, The Netherlands

**Keywords:** Neuroscience, Human behaviour

## Abstract

There is convincing evidence from rodent studies suggesting that prenatal infections affect the offspring’s brain, but evidence in humans is limited. Here, we assessed the occurrence of common infections during each trimester of pregnancy and examined associations with brain outcomes in adolescent offspring. Our study was embedded in the Generation R Study, a large-scale sociodemographically diverse prospective birth cohort. We included 1094 mother-child dyads and investigated brain morphology (structural MRI), white matter microstructure (DTI), and functional connectivity (functional MRI), as outcomes at the age of 14. We focused on both global and focal regions. To define prenatal infections, we composed a score based on the number and type of infections during each trimester of pregnancy. Models were adjusted for several confounders. We found that prenatal infection was negatively associated with cerebral white matter volume (B = −0.069, 95% CI −0.123 to −0.015, *p* = 0.011), and we found an association between higher prenatal infection scores and smaller volumes of several frontotemporal regions of the brain. After multiple testing correction, we only observed an association between prenatal infections and the caudal anterior cingulate volume (B = −0.104, 95% CI −0.164 to −0.045, *p* < 0.001). We did not observe effects of prenatal infection on other measures of adolescent brain morphology, white matter microstructure, or functional connectivity, which is reassuring. Our results show potential regions of interest in the brain for future studies; data on the effect of severe prenatal infections on the offspring’s brain in humans are needed.

## Introduction

Common infections during pregnancy usually do not have major effects on the health of the mother, but there has been long-standing concern if, and to what extent, prenatal infections might have an impact on the child [1–6]. The need to address these questions has been intensified by the SARS-CoV-2 pandemic, as millions of pregnant women worldwide have been infected with the virus, with potential implications for fetal brain development [4]. Although associations between common infections during pregnancy and increased risks of psychopathology in offspring have been observed in multiple, large-scale register-based studies [1, 7–10], both the causal link as well as the effect on brain structure- and function later in life are not evident [3].

There is abundant evidence from rodent studies that lipopolysaccharides (LPS; bacterial toxins), polyinosinic-polycytidylic acid (poly I:C; simulating a viral infection), and influenza (induced with H1N1 or H3N2), lead to profound structural brain abnormalities in rodent offspring [2, 3, 11–13], including reductions in intracranial volume, gray matter volume, and white matter volume [2, 14]. Specifically, studies demonstrate reductions in cortical gray matter volume and white matter volume in adolescent mice [11], smaller hippocampal volumes in neonatal mice [15], and alterations in neurocellular responses such as gliosis in both neonatal and adolescent mice [12, 16]. Yet, these effects cannot be directly extrapolated to the human brain for multiple reasons, including the distinct timeline of brain development in humans compared to rodent offspring [17]. In humans, very little is known about the prospective association between prenatal infections and offspring brain outcomes.

Fetal brain development follows a tightly regulated cascade of events, which can be influenced by the mother’s health during pregnancy [1, 3, 18]. Experimental studies show that the environmental risk factor ‘prenatal infection’ may affect the child’s brain in various ways. Prenatal infection, and the subsequent activation of the mother’s immune system may lead to placental inflammation, and consequently an increase in pro-inflammatory cytokines in both mother and fetus. This may affect the fetal brain by either dysregulating placental serotonin signaling, microglial priming, fetal brain neurotransmitter signaling, or by disrupting mitochondria and protein homeostasis leading to oxidative stress [4, 18–21]. The effect of prenatal infection on the child’s brain may also be caused via vertical transmission, i.e., the pathogen infects the fetus by transfer over the placenta and as such leads to a pathogen-specific response in the fetal brain [3, 6], of which several examples exist including Zika-virus, Toxoplasma Gondii, or Rubella virus [22].

As there are limited human studies investigating the effect of prenatal exposure to infections on the offspring’s brain, the primary aim of our current study is to investigate the potential long-term association of prenatal infection at each trimester of pregnancy with brain outcomes in adolescence. We used a composite score to measure common prenatal infections in each trimester of pregnancy as the exposure. Given that we are using data of a large-scale population-based birth cohort with more than 1000 mother-child dyads, we followed recommendations of a recent brain-wide association study [23]. Hence, we used a comprehensive approach i) to get a broader understanding if and to what extent the brain is affected at multiple levels, and ii) for the purpose to generate hypotheses for future advancements in the field. To do so, we examined different modalities of the brain, including brain morphology, white matter microstructure, network efficiency and functional connectivity, as outcomes. Considering the high prevalence of common infections, we applied stringent multiple testing correction to reduce the risk of false positives, as to not potentially unnecessarily worry pregnant women. This means that we do increase the risk of false negative findings, which could also be potential targets in the brain; therefore, we highlight the effect sizes and confidence intervals in each association. We hypothesized global effects on the brain, and in secondary analyses we also explored focal effects on the brain. Moreover, we investigated the effect of the presence of fever (as fever is a sign that a particular infection leads to a more pronounced and systemic inflammatory response), the effect of timing of infection (i.e., detecting potential trimester-specific effects), and whether the associations are moderated by sex of the child.

## Methods

### Study selection and participants

This study was embedded in the Generation R Study, a large prospective population-based cohort investigating the development of maternal and child health from fetal life onwards [24–26]. Pregnant women living in Rotterdam, the Netherlands, were initially recruited between April 2002 and January 2006. In the neuroimaging research visit when the child was 13–16 years old, a total of 3571 children participated. To be included in the current study, mothers had to have been enrolled in the first trimester of their pregnancy, and the data on infection during pregnancy as well as on one of the three adolescent brain imaging modalities had to be available. We randomly excluded one twin or sibling from each sibling pair. In addition, we excluded children with incomplete data, dental braces, poor image quality or incidental findings on the scan, which interfere with the reconstruction of the images, or which significantly alter brain morphology. The Medical Ethics Committee of the Erasmus Medical Centre approved all study procedures. All parents provided written informed consent and children provided assent. More details about the Generation R study design can be found elsewhere [27].

### Prenatal infection assessment

To define prenatal infections, we used prospective questionnaire data collected at three time points during pregnancy. Women were asked to report on the following infection items: upper respiratory infections (pharyngitis, rhinitis, sinusitis, ear infection), lower respiratory infections (pneumonia, bronchitis), gastrointestinal infections (diarrhea, enteritis), cystitis/pyelitis, dermatitis (boils, erysipelas), eye infections, herpes zoster, flu, sexually transmitted diseases (STD), and a period of fever ( > 38 °C/100.4 °F) within the past two (asked during second trimester) or three months (asked during first and third trimesters). Figure S1 shows the distribution of each infection type per trimester.

Using a two-step approach, we constructed four sum scores: one for each trimester (trimester-based) and one spanning pregnancy. For the trimester-based sum score, each confirmation of the presence of a condition within that trimester (‘yes’) was scored as one point. A ‘no’ response was rated with 0 points. Consequently, a total sum score of 30 points could be derived for the whole pregnancy for women with trimester-based sum scores available at all three time points (10 points per trimester). In addition, we used fever as a separate severity marker. Accordingly, we constructed a binary variable categorizing fever as ‘yes’ and ‘no’ for each trimester and one sum score spanning pregnancy. For the sum score, one point was given for the presence of fever per trimester, resulting in a maximum sum score of 3 per pregnancy. Within the Generation R cohort there is information on C-reactive protein (CRP) levels in early pregnancy at a fixed single time point but not during the occurrence of infection. Given the lack of correlation between CRP and infection (see Figure S2), we did not include CRP in the infection score.

### Brain assessment

We used three different MRI modalities to examine the brain. To obtain information on brain morphology, white matter microstructure, and functional connectivity, we used structural magnetic resonance imaging (sMRI), diffusion tensor imaging (DTI), and resting state functional magnetic resonance imaging (fMRI), respectively. Since we had no hypothesis on lateralized effects, we combined the right and left hemisphere for each sMRI or DTI measure by summing up both hemispheres. Given the limited literature on the topic, we took both global (i.e., whole brain metrics) and focal (i.e., regional metrics) approaches.

#### Imaging acquisition

All adolescents (age range 13–16 years) were offered to participate in a mock MRI scanning session to become familiar with the MRI procedure [28]. A 3-Tesla GE Discovery MR750w (GE, Milwaukee, WI) system was used [29]. High resolution T1-weighted scans were obtained with an IR-FSPGR sequence with the following parameters: repetition time=8.77 ms, echo time=3.4 ms, inversion-time = 600 ms, number of excitations = 1, flip angle = 10°, acquisition matrix size = 220 × 220, ARC acceleration factor=2, slices = 230, and in-plane resolution = 1.0 mm^3^ [28]. The DTI measurement included a 35-direction echo planar imaging sequence, with the following parameters: repetition time=12,500 ms, echo time = 72 ms, field of view = 240 mm × 240 mm, acquisition matrix = 120 × 120, slice thickness = 2 mm, slices = 65, ASSET acceleration factor=2, b = 900 s/mm^2^, 3 b = 0 images. The fMRI measurement included 200 volumes of data with an interleaved axial echo planar imaging sequence, with the following parameters: repetition time = 1760 ms, echo time = 30 ms, flip angle = 85°, matrix = 64 × 64, field of view = 230 × 230 mm, and slice thicknes = 4 mm [28]. The fMRI scan lasted 5 min 52 s during which the adolescents were asked to stay awake and keep their eyes closed.

#### Image processing

sMRI data were processed with the FreeSurfer analysis suite [30]. DTI data were processed with a fully automated probabilistic fiber tractography from which subject-specific probabilistic representations of different white matter fibers bundles were obtained (FSL plugin ‘AutoPtx’ (http://fsl.fmrib.ox.ac.uk/fsl/fslwiki/AutoPtx) [31, 32]. fMRI data was preprocessed with the FMRIPrep package (version 20.1.1) [33], and functional connectivity measures were obtained using the Gordon parcellation scheme [34]. A summary of the image processing and quality assurance for all three modalities can be found in the supplementary text, and full details can be found elsewhere [28–32].

#### Structural MRI

We studied global brain morphology, for which we included the following volumes: total brain, lateral ventricles, cerebellum, cortical gray matter, and cerebral white matter. In addition, we analyzed several subcortical volumes (brain stem, hippocampus, amygdala, thalamus, accumbens, caudate, putamen, corpus callosum posterior/mid posterior/central/mid anterior/anterior), and we included all cortical regional volumes from the Desikan-Killiany atlas [35].

#### DTI

To obtain information on white matter microstructure, we examined mean fractional anisotropy and mean diffusivity from the following tracts: uncinate fasciculus, cingulum bundle, superior longitudinal fasciculus, forceps minor, forceps major, inferior longitudinal fasciculus, and corticospinal tract. The anatomy and function of these tracts have been well-described in the literature [36]. We also included global fractional anisotropy (FA) and global mean diffusivity (MD), these are weighted (by tract volume) averages of all tracts. Higher FA indicates a preferential direction in water diffusion, implying more structure in the underlying white matter microstructure, whereas MD is inversely correlated with FA and; thus, displays the average diffusivity in all directions.

#### Functional MRI

Further, to represent functional network connectivity, we obtained graph theory measures (specifically, global efficiency, modularity, and characteristic path length) [37], and we obtained the within and between functional connectivity indices for the following thirteen networks: none, default, parieto occipital, frontoparietal, salience, cingulo opercular, medial parietal, dorsal attention, visual, somatomotor hand, somatomotor mouth, and auditory. Functional connectivity indicates the temporal relationship between functional networks in the brain [38]. Graph theory measures summarize functional connectivity characteristics across the brain and between networks [39]. Global efficiency attributes the capacity for integrated processing and parallel information transfer. Modularity quantifies to what magnitude networks can be partitioned into segregated communities. Characteristic path length computes the mean number of steps in the shortest paths connecting each pair of nodes. The between functional connectivity network is defined as the average functional connectivity between the specified network and the other networks.

### Covariates

We adjusted all models for multiple covariates. Age at scan was calculated based on the child’s date of birth and the date of the scan. Hospital registries provided information on child biological sex. Maternal national background (‘Dutch’ or ‘non-Dutch’) was established via the enrollment questionnaire. Moreover, maternal age at enrollment, maternal tobacco use (‘no’, ‘yes, until pregnancy was known,’ and ‘yes, continued during pregnancy’), maternal alcohol consumption (‘none during pregnancy,’ ‘drank until pregnancy was known,’ ‘continued drinking occasionally’ (less than one glass per week), and ‘continued drinking frequently’ (one or more glass/week for at least two trimesters), drug use (i.e., marijuana, hashish, cocaine, heroin, or ecstasy) during pregnancy (‘no’, ‘yes, until pregnancy was known,’ and ‘yes, continued during pregnancy’), were prospectively assessed with a questionnaire at enrollment. In addition, household income and maternal education were assessed via questionnaires at enrollment. Prenatal maternal psychopathology was measured with a validated self-reported questionnaire (Brief Symptom Inventory) [40]. From this 53-item questionnaire, a Global Severity Index was calculated and used as a continuous score, with higher scores indicating more problems. Household income was divided into two categories based on the Dutch net average income in the enrollment years (2000–2006): ‘<€2200/month’ and ‘>€2200/month’. Based on Statistics Netherlands classifications, three categories were created for parental education: ‘primary’ (no education or primary school), ‘intermediate’ (secondary school or lower vocational training) and ‘high’ (higher vocational training or university). Intracranial volume was measured using sMRI when the child was 13–16 years old.

### Statistical analyses

All analyses were conducted in the R Statistical Software (version 3.6.1). We applied a non-response analysis to explore if there were biases due to the selection that took place in forming the sub-sample for this analysis. Categorical variables were compared with the Chi-square test and continuous variables were compared with a two-sample T-test. To account for missing data on covariates (maximum missingness≈7%), we applied multiple imputation using chained equations with the ‘mice’ package in R, and we imputed 30 iterations and 30 datasets [41].

**Fig. 1:**
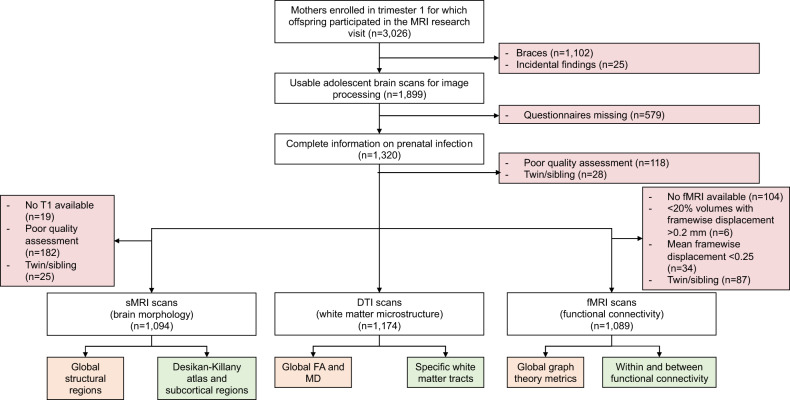
Flowchart study population with global and focal brain outcomes for all three modalities (sMRI, DTI, and fMRI). The exclusion criteria are indicated with red. The global measures are indicated with orange and the focal measures are indicated with green. Global sMRI measures include volumes of total brain, lateral ventricles, cerebellum, cortical gray matter, and cerebral white matter. Focal sMRI measures include all the brain regions from the Desikan-Killany atlas, and subcortical regions such as the brain stem, caudate, hippocampus, amygdala, putamen, amygdala, thalamus, accumbens, and corpus callosum (posterior/ mid posterior/ central/ mid anterior/ anterior). Global DTI measures include global mean diffusivity and global fractional anisotropy. Focal DTI measures include the fractional anisotropy (FA) and mean diffusivity (MD) of seven previously described white matter tracts. Global fMRI measures include global graph theory measures, such as global efficiency, modularity, and characteristic path length. Focal fMRI measures include within and between functional connectivity matrices for thirteen previously described networks.

We standardized the prenatal infection sum score and the brain outcomes to Z-scores (mean = 0, SD = 1) to enable comparison between the outcomes. We used multiple linear regression analyses, with the prenatal infection sum score as exposure and the three imaging modalities of the adolescent brain (sMRI, DTI and fMRI measures) as outcomes. Given the limited literature, for each modality, we took both a global (hypothesis-based) and a focal (exploratory-based) approach (Fig. 1).

We investigated the effect of the presence of fever using the fever sum score as exposure and repeating the above-mentioned linear regressions for the significant global or focal brain regions. Moreover, we repeated the linear regressions using trimester-based sum scores for prenatal infection as exposure to investigate the effect of timing of infection for the significant global or focal brain regions. Lastly, to investigate if the association between prenatal infection and significant brain regions was sex-specific, we investigated biological sex as moderator in those associations by adding an interaction term between prenatal infection and child sex.

Models were initially adjusted for all covariates (age of the mother, national background of the mother, household income, maternal education, maternal psychopathology, age of the child, sex of the child, and intracranial volume). A second model was then fitted with the same covariates, but without intracranial volume, to better understand the complete picture of associations.

We calculated the effective numbers of tests for the global and focal outcomes separately and used them to correct for multiple testing according to the Bonferroni method [42, 43]. This resulted in 7.5 and 47.1 as the number of effective tests for the global and focal outcomes, respectively (see also the correlation matrices in Figures S3 and S4). Accordingly, the corrected significance thresholds were p-values below 0.007 and 0.001 (instead of 0.05) for the global and focal outcomes, respectively.

## Results

### Demographics participants

The final sMRI sample was 1094 mother-child pairs, after applying the inclusion and exclusion criteria for the primary analysis (Fig. 1). The baseline characteristics of the participants are represented in Table 1. In general, included mothers, as compared to the excluded sample from the baseline Generation R cohort, were older (mean difference = 1.69 years, df = 1412, *p* < 0.001), had higher income (χ^2^ = 101, df = 1, *p* < 0.001), were more often of Dutch national background (χ^2^ = 119, df = 1, *p* < 0.001), and had a higher education (χ^2^ = 135, df = 2, *p* < 0.001). Sample sizes were slightly different for DTI and fMRI samples; however, importantly, non-response analyses showed a similar pattern across all samples.

**Table 1:** Baseline characteristics.

	sMRI sample (*n* = 1094)
**Maternal characteristics**	
Age mother at enrollment (mean, SD)	31.3 (4.4)
Caesarian delivery (*n*, %)	118 (10.9)
Parity (mean, SD)	0.5 (0.7)
*National background (n, %)*	
Dutch	710 (64.9)
Non-Dutch	377 (34.5)
*Maternal education (n, %)*	
Low	49 (4.5)
Intermediate	416 (38.0)
High	625 (57.1)
*Household income (n, %)*	
< €2000 per month	342 (31.3)
> €2000 per month	705 (64.4)
*Smoking habits (n, %)*	
Never smoked during pregnancy	820 (75.0)
Smoked until pregnancy was known	97 (8.9)
Continued smoking in pregnancy	164 (15.0)
*Alcohol consumption (n, %)*	
Never drank in pregnancy	354 (32.4)
Drank until pregnancy was known	182 (16.6)
Continued drinking occasionally	419 (38.3)
Continued drinking frequently	133 (12.2)
**Child characteristics**	***n*** = **1094**
Age child MRI (mean, SD)	14 (0.6)
Child’s sex, female (%)	586 (53.6)
Gestational age at birth (weeks) (mean, SD)	40 (1.6)
Birth weight (grams) (mean, SD)	3578 (545)

### Brain morphology

#### Primary analyses

**Fig. 2:**
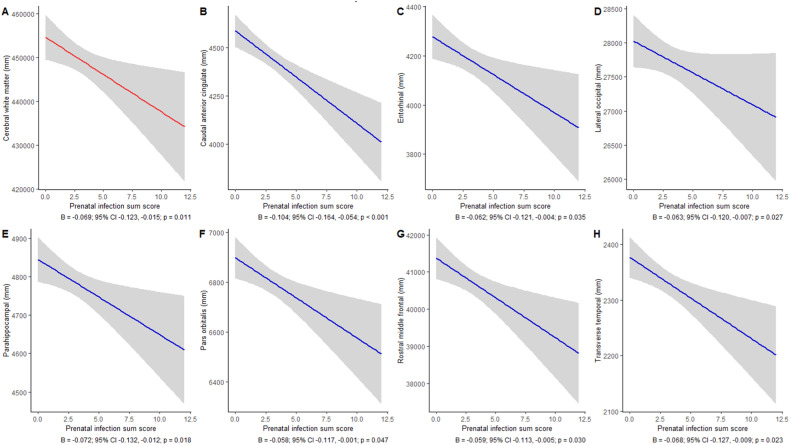
Prenatal infection and significant (p_uncorrected_ < 0.05) adolescent brain morphology regions. **A**–**H** shows the regression plots for all significant global and focal regions including the effect estimates. To distinguish between global and focal regions, the effects in global regions are indicated with a red line and the effects in focal regions are indicated with a blue line. Below each graph the effect size, confidence interval and *p*-value are noted.

We did not observe an association between prenatal infection and intracranial volume. Moreover, we did not find an association between prenatal infection and volumes of the lateral ventricles, cerebellum, or cortical gray matter. We found a negative association between prenatal infection and cerebral white matter volume (B = −0.069, 95% CI −0.123, −0.015, *p* = 0.011), but this was not significant after correction for multiple testing (Table 2, Fig. 2A).

**Table 2:** Prenatal infection and global brain outcomes.

	β-coefficient	95% Confidence interval		*P*-value
**Global sMRI**				
Total brain	0.093	−0.086	0.273	0.306
Lateral ventricles	−0.038	−0.099	0.022	0.218
Cerebellum	0.004	−0.049	0.057	0.881
Cortical gray matter	−0.046	−0.097	0.004	0.072
Cerebral white matter	−0.069	−0.123	−0.015	0.011*
**Global DTI**				
Global mean fractional anisotropy	0.011	−0.047	0.070	0.696
Global mean diffusivity	0.000	−0.059	0.059	0.998
**Global fMRI**				
Global efficiency	−0.016	−0.080	0.047	0.605
Modularity	−0.003	−0.066	0.059	0.906
Characteristic path length	0.011	−0.051	0.074	0.718

*p < 0.05.

**Fig. 3:**
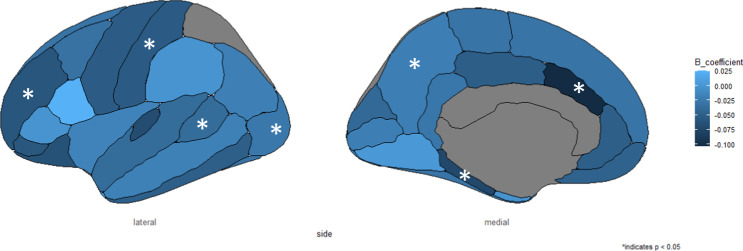
Results of all cortical brain regions. The starred regions entail the significant cortical regions, namely the volumes of entorhinal, caudal anterior cingulate, lateral occipital, parahippocampal, pars orbitalis, rostral middle frontal, and transverse temporal.

For the focal regions, we observed negative associations between prenatal infection and entorhinal volume (B = −0.062, 95% CI −0.121, −0.004, *p* = 0.035), caudal anterior cingulate volume (B = −0.104, 95% CI −0.164, −0.045; *p* < 0.001), lateral orbitofrontal volume (B = −0.063, 95% CI −0.120, −0.007, *p* = 0.027), parahippocampal volume (B = −0.072, 95% CI −0.132, −0.012, *p* = 0.018), pars orbitalis volume (B = −0.058, 95% CI −0.117, −0.001, *p* = 0.047), rostral middle frontal volume (B = −0.059, 95% CI −0.113, −0.005, *p* = 0.030), and transverse temporal volume (B = −0.068, 95% CI −0.127, −0.009, *p* = 0.023). After multiple testing correction, we only observed the negative association between prenatal infection and the caudal anterior cingulate volume (Table S1, Figs. 2B–H and 3).

Even though prenatal infection was not associated with total brain volume, we repeated all analyses with adjustment for intracranial volume and results were similar, but effect sizes were slightly attenuated as expected (Tables S2 and S3).

#### Secondary analyses

We observed no moderating effect of fever, timing of infection or sex after correcting for multiple testing (Table S4–S6). A negative association between fever and caudal anterior cingulate volume (B = −0.058, 95% CI −0.110, −0.006, *p* = 0.026) was observed before multiple testing correction. Moreover, a negative association was observed between first trimester prenatal infection and caudal anterior cingulate volume (B = −0.057, 95% CI −0.110, −0.005, *p* = 0.031) before multiple testing correction. Negative associations were observed for second trimester prenatal infection and caudal anterior cingulate volume (B = −0.077, 95% CI −0.130, −0.024, *p* = 0.004) and cerebral white matter volume (B = −0.029, 95% CI −0.059, 0.000, *p* = 0.048) before multiple testing correction. We further observed a positive association between second trimester prenatal infection and accumbens volume (B = 0.061, 95% CI 0.009, 0.114, *p* = 0.020) before multiple testing corrections.

### White matter microstructure

We observed no association of prenatal infection on adolescent white matter microstructure. Specifically, we found no association with global fractional anisotropy (B = 0.011, 95% CI −0.047, 0.070, *p* = 0.696) and global mean diffusivity (B = 0.000, 95% CI −0.059, 0.059, *p* = 0.998) (Table 2). We further observed no association between prenatal infection and the fractional anisotropy or mean diffusivity of the following tracts: uncinate fasciculus, cingulum, superior longitudinal fasciculus, forceps minor, forceps major, inferior longitudinal fasciculus, and corticospinal tract (Table S7).

### Network efficiency and functional connectivity

We observed no association of prenatal infection on measures of functional network connectivity. Specifically, we observed no association between prenatal infection and global efficiency (B = −0.016, 95% CI −0.080, 0.047, *p* = 0.605), modularity (B = −0.003, 95% CI −0.066, 0.059, *p* = 0.906), and characteristic path link (B = 0.011, 95% CI −0.051, 0.074, *p* = 0.718) (Table 2). We further did not observe an association between prenatal infection and the within and between functional connectivity of the previously described thirteen networks (Table S8).

Given our structural findings in the caudal anterior cingulate, we applied a post-hoc analysis to investigate whether the functional connectivity of the caudal anterior cingulate cortex (calculated based on four regions of the Gordon Parcellation scheme) associates with the other 329 regions of the Gordon Parcellation scheme and the Freesurfer subcortical segmentation. None of the associations survived multiple testing correction (Supplementary Table S9).

## Discussion

In this large population-based cohort study, we studied global effects of common infections during pregnancy on the offspring’s brain. We found a negative association between prenatal infections and cerebral white matter volume, which is of interest given that preclinical studies showed white matter damage after in utero exposure to infections [18], but this finding was not significant after correction for multiple testing. In addition, we did not find associations between prenatal infection and white matter microstructure, network efficiency, and functional connectivity. Moreover, and in contrast to the animal literature, we did not find reductions in total brain volume or gray matter volume [2, 3, 11–13].

In the focal brain regions, we did observe an association between higher prenatal infection scores and smaller volumes of several frontotemporal regions of the brain such as entorhinal, lateral occipital, parahippocampal, pars orbitalis, rostral middle frontal, and transverse temporal regions, but after stringent corrections for multiple testing, this was only significant for the association between prenatal infection and a decreased caudal anterior cingulate volume. The caudal anterior cingulate is a region of interest, as it is part of the anterior cingulate cortex and as such linked to executive functioning in the frontal lobe, sensorimotor systems in the parietal lobe, and emotional processes in the limbic lobe [44]. Moreover, the caudal anterior cingulate region has been related to various psychiatric disorders [45–47]. For example, a decrease in volume in the caudal anterior cingulate region has been related to depression [48], positive symptoms in schizophrenia [49], higher polygenic risk scores for anxiety disorders [50], and first-onset psychosis [51]. Therefore, it is tempting to speculate that our finding in the caudal anterior cingulate after in utero exposure to common infections could be associated with higher risks of psychiatric disorders.

We report our findings after rigorous correction for multiple testing, which is the consequence of the explorative design of our study including multiple brain modalities in one study. We wanted to reduce the risk of false positive findings, given that this would potentially cause unneeded concern in a vulnerable group: pregnant individuals who are frequently diagnosed with common infections (e.g., upper respiratory, gastrointestinal, or urinary tract infections). While our adjusted significance threshold has reduced the risk of false positives, which is favorable for the reproducibility of our findings, this approach also increased the risk of false negatives and lower statistical power. This paradox has been well described by a group of MRI experts in a recent large brain-wide association study [23]. In addition, the effect sizes of these frontotemporal regions, while slightly different from the caudal anterior cingulate, show similar patterns across different levels of the prenatal infection sum score. The main difference between these regions and the caudal anterior cingulate are the wider confidence intervals. Therefore, future research could still include these frontotemporal regions as regions of interest.

Our study reports many negative findings, a plausible explanation could be that we investigated a range of mild infections, while the effects of prenatal infection on the offspring’s brain may be pathogen-specific and related to more severe infections. Therefore, there is a need for follow-up studies including pregnant women who have been admitted for infections and their offspring, to specifically investigate the risk after severe infections. Another explanation for our mainly negative findings could be that effects of prenatal infection may occur on a more molecular signaling pathway level, as suggested by rodent studies in which transcriptomic changes were found [52, 53]. This may not be detected with sMRI, DTI, or fMRI, given the relatively coarse resolution. In addition, recent preclinical studies demonstrated altered dendritic morphology in the prefrontal cortex after exposure to prenatal infection [54, 55], which is also suggestive for neuronal morphological changes rather than gross volumetric changes. Moreover, a recent study demonstrated that a prenatal SARS-CoV-2 infection was associated with cortical hemorrhages in human fetal brain tissue, which was further associated with a decline in blood vessel integrity and infiltration of immune cells into the fetal brain [56]. Alternatively, there may be no substantial associations between common prenatal infections and the adolescent’s brain, which is reassuring given that mothers are frequently exposed to infections during pregnancy.

To the best of our knowledge, this is one of the first studies in humans to examine the relationship between common prenatal infections and offspring brain structure and function. Some prior evidence in rodents suggested that prenatal infections are associated with reductions in offspring hippocampal [15], intracranial, cortical gray and white matter volume [5, 11]. Notably, the development of the brain is different in humans compared to rodents [17], and the severity of infection may be another explanation for the discrepancies in these results. In most rodent studies, infection is evoked by LPS, poly:IC, or the influenza virus (such as H1N1 or H3N2). These immune inducers lead to severe systemic infections which differ from the broad spectrum of common infections we studied in our human study. Interestingly, there is some cross-sectional literature in rodents also reporting limited associations between prenatal infection and the structural brain abnormalities in the offspring’s brain [52, 57]. The age (adjusted) range in these animals was similar to the age range of the humans in our study, and while they observed clear behavioral problems in rodent offspring, neuroanatomical differences were limited, even after stratifying for behavioral deficits. A longitudinal rodent study showed that the association between prenatal infection and child brain development is age dependent. Specifically, changes (e.g., reductions in brain volume) were observed at an early age, but largely normalized at the time of adulthood [58]. This study was also the first to show that this association was also observed in mice offspring that were subjected to a prenatal infection model of high intensity. A subsequent preclinical study validated this finding, namely that alterations in brain volume after prenatal exposure to infections were of temporary nature [53]. Hence, early effects of infection on the brain may be transient, reflective of the inherent plasticity of the brain.

While limited previous work in humans has focused specifically on common prenatal infections, there is evidence that severe perinatal infections have an impact on the brain. There are some studies on chorioamnionitis, which is defined as inflammation of the placental issue due to a bacterial infection (often caused by the endogenous bacteria in the mother’s reproductive tract), after which the fetal membranes and amniotic fluid during labor and delivery are affected. Chorioamnionitis may lead to sepsis and meningitis in the neonate as well as brain injury such as hypoxic-ischemic encephalopathy [59–61]. Moreover, exposure to chorioamnionitis in preterm children has been found to be associated with widespread changes in cortical thickness in two-year-old children [62]. We do not have data on the occurrence of chorioamnionitis, but we expect the prevalence to be very low, given that we used a population-based sample.

In addition, there are a few human studies that have focused on exposure to elevated maternal inflammatory markers during pregnancy. These studies showed an association between increased levels of cytokine interleukin-6 (IL-6) and white matter microstructure changes around the amygdala in one-year old children (*N* = 86) [63], and structural and functional changes in the amygdala when these children were two years old (*n* = 86) [64]. In the same cohort, functional changes in several networks such as the salience, subcortical, dorsal attention, cerebellar, visual, frontoparietal and ventral attention network were found when the children were two-years old (*n* = 84) [65], and at age 5 years (*n* = 42), structural changes in the pars triangularis volume were reported [66]. Further, we previously reported a negative association between CRP in early pregnancy with cerebellar volume in 10-year-old children [67]. In all these previous studies IL-6 or CRP were not measured during infection, but at a fixed timepoint during pregnancy. Therefore, the association with infection is unclear given that both IL-6 and CRP are rapidly restored to the normal range once the infection is cleared. Next to infection, an upregulation of these immune markers is further associated with a wide range of somatic conditions, such as auto-immune disease or physical injury [68, 69], but also related to other factors, such as stress [70], obesity [71] and smoking [72]. In our study, we specifically investigated the association between the occurrence of common prenatal infections and the brain and therefore, comparability with these previous studies is very limited.

The strengths of this study include the use of a large socio-demographically diverse, prospective population-based cohort (N ≈ 1100), the investigation of a broad range of infections during each trimester of pregnancy, and the use of multiple neuroimaging modalities (sMRI, DTI, and fMRI) to investigate the adolescent’s brain. The study also has limitations which warrant discussion. First, information on prenatal infections was collected once per trimester and no blood was drawn at time of infection. Second, we used self-reported questionnaires; however, we consider recall bias unlikely given that infections were recalled after each trimester ( ~ 2–3 month recall) [73]. Moreover, self-report questionnaires, rather than a visit to the research center for biological measurements, are less prone to healthy volunteer bias, whereby participants are less likely to attend a research visit if they are infected. Yet, future research may benefit of serological evidence to confirm the presence of bacterial or viral infections. Third, as our study contains observational data, we cannot determine causal relationships nor determine if our results are either masked or driven by brain maturation. Fourth, some demographic variables were different between the included mothers and the baseline Generation R cohort, which may impact the generalizability of our findings. Yet, our sample is quite representative for the general Dutch pregnant population in terms of age, education, and lifestyle, and we adjusted for these variables. Fifth, the single time point nature of the imaging outcomes limited our ability to interpret and disentangle the precise nature of the associations between prenatal infection and the child’s brain. Lastly, even though we adjusted for confounders, the associations can still be the result of unmeasured or residual confounding.

In conclusion, we investigated the long-term association between prenatal infection and different modalities of the adolescent’s brain in the general population. We report some evidence for negative associations between common prenatal infections in mainly the caudal anterior cingulate volume and some trends in white matter volume and several frontotemporal regions. We further observe no other morphological, microstructural, or functional changes after stringent correction for multiple testing. Considering the abundance of animal studies suggesting brain abnormalities, we believe it is important to highlight these findings in the human brain. Our results show potential targets in the brain for future studies to get a better understanding if there are long-term effects of exposure to common prenatal infections on the offspring’s brain. Future studies could also extend to the effects of severe prenatal infections on the offspring’s brain in humans and include other neuroimaging modalities to get a more comprehensive understanding of potential effects on different levels of the brain.

### Supplementary information

Supplementary information

## Data Availability

Computer code used for this project is shared at: https://github.com/ajsuleri/prenatal_infection_multimodal_imaging.
