# Supplementary material for: Long-term effects of prenatal infection on the human brain: a prospective multimodal neuroimaging study
Source: Transl Psychiatry. 2023 Oct 3;13:306. doi: 10.1038/s41398-023-02597-x (PMC10547711; doi:10.1038/s41398-023-02597-x)
Supplement: Supplementary file 1 — Supplementary information [file 41398_2023_2597_MOESM1_ESM.docx]

**SUPPLEMENTARY CONTENT**

Table of Contents

[SUPPLEMENTARY TEXT 2](#_Toc130147964)

[sMRI 2](#_Toc130147965)

[Imaging processing 2](#_Toc130147966)

[Imaging quality assurance 2](#_Toc130147967)

[DTI 2](#_Toc130147968)

[Imaging processing 2](#_Toc130147969)

[Imaging quality assurance 2](#_Toc130147970)

[fMRI 2](#_Toc130147971)

[Imaging processing 2](#_Toc130147972)

[Imaging quality assurance 2](#_Toc130147973)

[References 3](#_Toc130147974)

[SUPPLEMENTARY TABLES 4](#_Toc130147975)

[**Table S1**. Prenatal infection and focal brain sMRI (not ICV corrected) (standardized coefficients) 4](#_Toc130147976)

[**Table S2**. Prenatal infection and global brain sMRI (intracranial volume corrected) (standardized coefficients) 5](#_Toc130147977)

[**Table S3**. Prenatal infection and focal sMRI outcomes (intracranial volume corrected) (standardized coefficients) 6](#_Toc130147978)

[**Table S4**. Effect of prenatal fever on significant brain outcomes (standardized coefficients) 7](#_Toc130147979)

[**Table S5**. Moderating effect of sex on significant brain outcomes (standardized coefficients) 8](#_Toc130147980)

[**Table S6**. Effect of timing of prenatal infection on significant brain outcomes (standardized coefficients) 9](#_Toc130147981)

[**Table S7.** Prenatal infection and focal DTI outcomes (standardized coefficients) 10](#_Toc130147982)

[**Table S8.** Prenatal infection and focal fMRI outcomes (standardized coefficients) 11](#_Toc130147983)

[**Table S9.** Region-based connectivity analysis 12](#_Toc130147984)

[SUPPLEMENTARY FIGURES 24](#_Toc130147985)

[**Figure S1.** Distribution different infection types. Figures 1A-C show the distribution of the infection types for trimester 1, 2 and 3, respectively. 24](#_Toc130147986)

[**Figure S2.** Correlation between C-reactive protein levels <18 weeks of gestation (1 fixed time point measurement available in Generation R) and total and trimester-based prenatal infection scores. 25](#_Toc130147987)

[**Figure S3**. Correlation plot global brain outcomes. 26](#_Toc130147988)

[**Figure S4**. Correlation plot focal brain outcomes. 27](#_Toc130147989)

# SUPPLEMENTARY TEXT

## sMRI

### Imaging processing

The data was processed with FreeSurfer analysis suite (version 6.0.0) [http://surfer.nmr.mgh.harvard.edu/]. The images were individually processed with FreeSurfer. Then, raw DICOM data was converted to ‘MGZ-files’, after which skull stripping, intensity normalization, and voxel segmentation of gray and white matter and cerebrospinal fluid took place. The Desikan-Killiany atlas was used to label the gray matter regions.^1^ More detailed steps can be found elsewhere.^2^

### Imaging quality assurance

To assure the quality of the scans, FreeSurfer image reconstructions were visually inspected by at least one rater (trained master, PhD, or postdoctoral students). The inspection entailed inspection of the gray-white matter delineating by FreeSurfer. All scans were rated on the Likert scale (poor, questionable, good). Unusably or poor-quality scans were excluded. The visual ratings were compared to an automated quality assessment and the Euler number. More details have been described previously.^3,4^

## DTI

### Imaging processing

Probabilistic fiber tractography was applied on each child’s native space diffusion data. This was performed with the FSL plugin ‘AutoPtx’. This plugin automatically identifies connectivity distributions for fiber bundles that are commonly reported. A nonlinear transformation from the FMRIB FA map was applied to generate connectivity distributions to map every child’s native space. The number of successful seed-to-target attempts was used to normalize connectivity distributions. More information can be found elsewhere.^5^

### Imaging quality assurance

Both manual and automated quality control was applied. For the manual assessment, visual inspected was performed using the sum-of-squares error of the tensor calculation and the tract reconstructions. For the automated assessment, translation and rotation motion parameters for the eddy tool was used in order to exclude data of children with excessive motion. More information can be found elsewhere.^5^

## fMRI

### Imaging processing

FMRIPrep pipeline (version 20.1.1) was used to pre-process all the images. This pipeline includes volume realignment for motion from rotation and translation, slice-timing correction, and inter-subject registration. Non-linear registration (antsRegistration tool, version 2.1.0) was used for spatial normalization. More information can be found elsewhere.^6^

### Imaging quality assurance

Scans with high motion (based on mean framewise displacement higher than 0.25 mm or more than 20% of the volumes with a framewise displacement higher than 0.2 mm) were excluded. Moreover, the scans were visually inspected for co-registration, major artifacts, and whole-brain coverage. More information can be found elsewhere.^7^

## References

1. Desikan RS, Ségonne F, Fischl B, et al. An automated labeling system for subdividing the human cerebral cortex on MRI scans into gyral based regions of interest. *NeuroImage*. 2006;31(3):968-980. doi:10.1016/j.neuroimage.2006.01.021

2. Fischl B. FreeSurfer. *NeuroImage*. 2012;62(2):774-781. doi:10.1016/j.neuroimage.2012.01.021

3. Muetzel RL, Blanken LME, van der Ende J, et al. Tracking Brain Development and Dimensional Psychiatric Symptoms in Children: A Longitudinal Population-Based Neuroimaging Study. *Am J Psychiatry*. 2018;175(1):54-62. doi:10.1176/appi.ajp.2017.16070813

4. White T, Muetzel RL, El Marroun H, et al. Paediatric population neuroimaging and the Generation R Study: the second wave. *Eur J Epidemiol*. 2018;33(1):99-125. doi:10.1007/s10654-017-0319-y

5. Dall’Aglio L, Xu B, Tiemeier H, Muetzel R. Longitudinal Associations Between White Matter Microstructure and Psychiatric Symptoms in Adolescence. *medRxiv*. Published online 2022. doi:https://doi.org/10.1101/2022.08.27.22279298

6. Dall’Aglio L, Estevez-Lopez F, Lopez-Vicente M, et al. Exploring the longitudinal associations of functional network connectivity and psychiatric symptom changes in youth. *medRxiv*. Published online 2022. doi:https://doi.org/10.1101/2022.11.26.22282787

7. López-Vicente M, Agcaoglu O, Pérez-Crespo L, et al. Developmental Changes in Dynamic Functional Connectivity From Childhood Into Adolescence. *Front Syst Neurosci*. 2021;15:724805. doi:10.3389/fnsys.2021.724805

# SUPPLEMENTARY TABLES

## **Table S1**. Prenatal infection and focal brain sMRI (not ICV corrected) (standardized coefficients)

|  | $\boldsymbol{\beta}$**-coefficient** | **95% Confidence interval** | | **P-value** |
| --- | --- | --- | --- | --- |
| Brain stem | -0.005 | -0.061 | 0.050 | 0.848 |
| Thalamus | -0.038 | -0.093 | 0.017 | 0.177 |
| Caudate | 0.008 | -0.050 | 0.067 | 0.784 |
| Putamen | 0.012 | -0.043 | 0.068 | 0.666 |
| Hippocampus | -0.023 | -0.080 | 0.033 | 0.414 |
| Amygdala | -0.026 | -0.081 | 0.029 | 0.359 |
| Accumbens | 0.055 | -0.004 | 0.114 | 0.067 |
| Banks of the superior temporal sulcus | -0.038 | -0.095 | 0.018 | 0.188 |
| Caudal anterior cingulate | -0.104 | -0.164 | -0.045 | <0.001** |
| Caudal middle frontal | -0.032 | -0.091 | 0.027 | 0.286 |
| Cuneus | -0.041 | -0.099 | 0.015 | 0.154 |
| Entorhinal | -0.062 | -0.121 | -0.004 | 0.035* |
| Fusiform gyrus | -0.030 | -0.084 | 0.024 | 0.276 |
| Inferior parietal | -0.019 | -0.074 | 0.035 | 0.484 |
| Inferior temporal | -0.050 | -0.106 | 0.004 | 0.072 |
| Isthmus cingulate | -0.027 | -0.081 | 0.027 | 0.329 |
| Lateral occipital | -0.026 | -0.080 | 0.027 | 0.337 |
| Lateral orbitofrontal | -0.063 | -0.120 | -0.007 | 0.027* |
| Lingual | 0.003 | -0.055 | 0.061 | 0.909 |
| Medial orbito frontal | -0.047 | -0.102 | 0.006 | 0.086 |
| Middle temporal | -0.018 | -0.073 | 0.036 | 0.514 |
| Parahippocampal | -0.072 | -0.132 | -0.012 | 0.018* |
| Paracentral | -0.030 | -0.087 | 0.026 | 0.296 |
| Pars opercularis | 0.025 | -0.033 | 0.084 | 0.394 |
| Pars orbitalis | -0.058 | -0.117 | -0.001 | 0.047* |
| Pars triangularis | -0.001 | -0.058 | 0.056 | 0.969 |
| Pericalcarine | -0.020 | -0.081 | 0.040 | 0.504 |
| Postcentral | -0.054 | -0.110 | 0.001 | 0.055 |
| Posterior cingulate | -0.051 | -0.107 | 0.005 | 0.074 |
| Precentral | -0.053 | -0.108 | 0.002 | 0.062 |
| Precuneus | -0.020 | -0.073 | 0.032 | 0.447 |
| Rostral anterior cingulate | -0.050 | -0.108 | 0.006 | 0.082 |
| Rostral middle frontal | -0.059 | -0.113 | -0.005 | 0.030* |
| Superior frontal | -0.030 | -0.087 | 0.020 | 0.220 |
| Superior parietal | -0.038 | -0.094 | 0.017 | 0.177 |
| Superior temporal | -0.035 | -0.093 | 0.022 | 0.229 |
| Supramarginal | -0.001 | -0.056 | 0.053 | 0.959 |
| Frontal pole | -0.026 | -0.087 | 0.034 | 0.388 |
| Temporal pole | 0.002 | -0.058 | 0.063 | 0.935 |
| Transverse temporal | -0.068 | -0.127 | -0.009 | 0.023* |
| Insula | -0.016 | -0.071 | 0.037 | 0.546 |
| Corpus callosum posterior | -0.014 | -0.075 | 0.046 | 0.650 |
| Corpus callosum mid posterior | -0.041 | -0.102 | 0.019 | 0.183 |
| Corpus callosum central | 0.020 | -0.041 | 0.081 | 0.518 |
| Corpus callosum mid anterior | -0.029 | -0.091 | 0.031 | 0.338 |
| Corpus callosum anterior | -0.057 | -0.119 | 0.003 | 0.066 |
| *p < 0.05  **Bonferroni-corrected p < 0.007 for global outcomes  **Bonferroni-corrected p < 0.001 for focal outcomes | | | | |

## **Table S2**. Prenatal infection and global brain sMRI (intracranial volume corrected) (standardized coefficients)

|  |  | $\boldsymbol{\beta}$**-coefficient** | **95% Confidence interval** | | **P-value** |
| --- | --- | --- | --- | --- | --- |
| **sMRI** |  |  |  |  |  |
|  | Lateral ventricles | -0.020 | -0.076 | 0.036 | 0.485 |
|  | Cerebellum | 0.026 | -0.019 | 0.071 | 0.263 |
|  | Cortical gray matter | -0.014 | -0.042 | 0.014 | 0.335 |
|  | Cerebral white matter | -0.034 | -0.064 | -0.004 | 0.024* |
| *p < 0.05  **Bonferroni-corrected p < 0.007 | | | | | |

## **Table S3**. Prenatal infection and focal sMRI outcomes (intracranial volume corrected) (standardized coefficients)

|  | $\boldsymbol{\beta}$ **-coefficient** | **95% Confidence interval** | | **P-value** |
| --- | --- | --- | --- | --- |
| Brain stem | 0.020 | -0.024 | 0.065 | 0.366 |
| Thalamus | -0.008 | -0.049 | 0.031 | 0.666 |
| Caudate | 0.031 | -0.019 | 0.082 | 0.230 |
| Putamen | 0.031 | -0.018 | 0.082 | 0.216 |
| Hippocampus | -0.003 | -0.050 | -0.045 | 0.915 |
| Amygdala | -0.003 | -0.051 | 0.043 | 0.877 |
| Accumbens | 0.075 | 0.022 | 0.128 | 0.005* |
| Banks of the superior temporal sulcus | -0.018 | -0.069 | 0.032 | 0.475 |
| Caudal anterior cingulate | -0.084 | -0.137 | -0.030 | 0.002* |
| Caudal middle frontal | -0.008 | -0.059 | 0.042 | 0.739 |
| Cuneus | -0.026 | -0.080 | 0.027 | 0.332 |
| Entorhinal | -0.051 | -0.108 | 0.005 | 0.074 |
| Fusiform gyrus | -0.004 | -0.048 | 0.038 | 0.823 |
| Inferior parietal | 0.004 | -0.041 | 0.050 | 0.862 |
| Inferior temporal | -0.026 | -0.072 | 0.019 | 0.258 |
| Isthmus cingulate | -0.003 | -0.048 | 0.041 | 0.875 |
| Lateral occipital | -0.003 | -0.048 | 0.041 | 0.879 |
| Lateral orbitofrontal | -0.037 | -0.082 | 0.007 | 0.105 |
| Lingual | 0.022 | -0.030 | 0.075 | 0.403 |
| Medial orbito frontal | -0.023 | -0.068 | 0.021 | 0.299 |
| Middle temporal | 0.006 | -0.038 | 0.051 | 0.777 |
| Parahippocampal | -0.053 | -0.108 | 0.001 | 0.055 |
| Paracentral | -0.008 | -0.058 | 0.041 | 0.734 |
| Pars opercularis | 0.045 | -0.007 | 0.098 | 0.094 |
| Pars orbitalis | -0.040 | -0.094 | 0.012 | 0.132 |
| Pars triangularis | 0.014 | -0.039 | 0.067 | 0.607 |
| Pericalcarine | -0.007 | -0.065 | 0.050 | 0.805 |
| Postcentral | -0.029 | -0.075 | 0.016 | 0.205 |
| Posterior cingulate | -0.026 | -0.073 | 0.019 | 0.257 |
| Precentral | -0.027 | -0.071 | 0.017 | 0.232 |
| Precuneus | 0.005 | -0.034 | 0.046 | 0.775 |
| Rostral anterior cingulate | -0.025 | -0.072 | 0.021 | 0.291 |
| Rostral middle frontal | -0.033 | -0.075 | 0.008 | 0.117 |
| Superior frontal | -0.006 | -0.046 | 0.034 | 0.765 |
| Superior parietal | -0.013 | -0.058 | 0.032 | 0.572 |
| Superior temporal | -0.008 | -0.054 | 0.037 | 0.718 |
| Supramarginal | 0.023 | -0.021 | 0.067 | 0.307 |
| Frontal pole | -0.020 | -0.080 | 0.040 | 0.512 |
| Temporal pole | 0.008 | -0.052 | 0.068 | 0.790 |
| Transverse temporal | -0.047 | -0.100 | 0.005 | 0.077 |
| Insula | 0.008 | -0.035 | 0.052 | 0.699 |
| Corpus callosum posterior | 0.0001 | -0.057 | 0.058 | 0.975 |
| Corpus callosum mid posterior | -0.026 | -0.084 | 0.031 | 0.373 |
| Corpus callosum central | 0.024 | -0.036 | 0.086 | 0.425 |
| Corpus callosum mid anterior | -0.023 | -0.084 | 0.036 | 0.439 |
| Corpus callosum anterior | -0.037 | -0.092 | 0.018 | 0.189 |
| *p < 0.05  **Bonferroni-corrected p < 0.001 | | | | |

## **Table S4**. Effect of prenatal fever on significant brain outcomes (standardized coefficients)

|  | $\boldsymbol{\beta}$ **-coefficient** | **95% Confidence interval** | | **P-value** |
| --- | --- | --- | --- | --- |
| Caudal anterior cingulate | -0.058 | -0.110 | -0.006 | 0.026* |
| Cerebral white matter | -0.010 | -0.039 | 0.019 | 0.499 |
| Accumbens | 0.040 | -0.011 | 0.091 | 0.127 |
| *p < 0.05  **Bonferroni-corrected p < 0.007 for cerebral white matter  **Bonferroni-corrected p < 0.001 for caudal anterior cingulate and accumbens | | | | |

## **Table S5**. Moderating effect of sex on significant brain outcomes (standardized coefficients)

|  | $\boldsymbol{\beta}$ **-coefficient** | **95% Confidence interval** | | **P-value** |
| --- | --- | --- | --- | --- |
| Caudal anterior cingulate | -0.060 | -0.138 | 0.017 | 0.130 |
| Cerebral white matter | -0.022 | -0.065 | 0.021 | 0.323 |
| Accumbens | 0.047 | -0.030 | 0.124 | 0.231 |
| *p < 0.05  **Bonferroni-corrected p < 0.007 for cerebral white matter  **Bonferroni-corrected p < 0.001 for caudal anterior cingulate and accumbens | | | | |

## **Table S6**. Effect of timing of prenatal infection on significant brain outcomes (standardized coefficients)

|  |  | $\boldsymbol{\beta}$ **-coefficient** | **95% Confidence interval** | | **P-value** |
| --- | --- | --- | --- | --- | --- |
| **Trimester 1** |  |  |  |  |  |
|  | Caudal anterior cingulate | -0.057 | -0.110 | -0.005 | 0.031* |
|  | Cerebral white matter | -0.025 | -0.054 | 0.004 | 0.096 |
|  | Accumbens | 0.051 | 0.000 | 0.104 | 0.051 |
| **Trimester 2** |  |  |  |  |  |
|  | Caudal anterior cingulate | -0.077 | -0.130 | -0.024 | 0.004* |
|  | Cerebral white matter | -0.029 | -0.059 | 0.000 | 0.048* |
|  | Accumbens | 0.061 | 0.009 | 0.114 | 0.020* |
| **Trimester 3** |  |  |  |  |  |
|  | Caudal anterior cingulate | -0.043 | -0.096 | 0.008 | 0.103 |
|  | Cerebral white matter | -0.018 | -0.048 | 0.010 | 0.216 |
|  | Accumbens | 0.046 | -0.005 | 0.099 | 0.080 |
| *p < 0.05  **Bonferroni-corrected p < 0.007 for cerebral white matter  **Bonferroni-corrected p < 0.001 for caudal anterior cingulate and accumbens | | | | | |

## **Table S7.** Prenatal infection and focal DTI outcomes (standardized coefficients)

|  | $\boldsymbol{\beta}$ **-coefficient** | **95% Confidence interval** | | **P-value** |
| --- | --- | --- | --- | --- |
| **Mean diffusivity** |  |  |  |  |
| Uncinate fasiculus | -0.006 | -0.065 | 0.053 | 0.840 |
| Cingulum | 0.014 | -0.045 | 0.074 | 0.636 |
| Superior longitudinal fasciculus | 0.000 | -0.058 | 0.059 | 0.995 |
| Forceps minor | -0.009 | -0.069 | 0.050 | 0.762 |
| Forceps major | 0.041 | -0.017 | 0.1014 | 0.167 |
| Inferior longitudinal fasiculus | -0.031 | -0.090 | 0.026 | 0.287 |
| Corticospinal tract | -0.020 | -0.080 | 0.039 | 0.505 |
| **Fractional anisotropy** |  |  |  |  |
| Uncinate fasiculus | 0.013 | -0.045 | 0.072 | 0.663 |
| Cingulum | -0.002 | -0.060 | 0.056 | 0.946 |
| Superior longitudinal fasciculus | -0.005 | -0.064 | 0.053 | 0.856 |
| Forceps minor | -0.012 | -0.071 | 0.047 | 0.683 |
| Forceps major | -0.008 | -0.068 | 0.051 | 0.783 |
| Inferior longitudinal fasiculus | 0.016 | -0.042 | 0.075 | 0.580 |
| Corticospinal tract | 0.030 | -0.029 | 0.089 | 0.316 |
| *p < 0.05  **Bonferroni-corrected p < 0.001 | | | | |

## **Table S8.** Prenatal infection and focal fMRI outcomes (standardized coefficients)

|  | $\boldsymbol{\beta}$ **-coefficient** | **95% Confidence interval** | | **P-value** |
| --- | --- | --- | --- | --- |
| **Within network functional connectivity** |  |  |  |  |
| None | -0.000 | -0.062 | 0.061 | 0.995 |
| Default | 0.016 | -0.045 | 0.077 | 0.602 |
| Parieto occipital | 0.001 | -0.060 | 0.063 | 0.963 |
| Frontoparietal | -0.038 | -0.101 | 0.024 | 0.226 |
| Salience | 0.017 | -0.046 | 0.081 | 0.586 |
| Cingulo opercular | -0.001 | -0.064 | 0.061 | 0.968 |
| Medial parietal | -0.032 | -0.096 | 0.031 | 0.314 |
| Dorsal attention | -0.029 | -0.091 | 0.033 | 0.358 |
| Ventral attention | 0.011 | -0.051 | 0.074 | 0.721 |
| Visual | -0.027 | -0.089 | 0.034 | 0.385 |
| Somatomotor hand | -0.022 | -0.086 | 0.040 | 0.479 |
| Somatomotor mouth | 0.005 | -0.058 | 0.068 | 0.871 |
| Auditory | 0.000 | -0.062 | 0.063 | 0.987 |
| **Between network functional connectivity** |  |  |  |  |
| None | -0.017 | -0.080 | 0.046 | 0.595 |
| Default | 0.014 | -0.049 | 0.078 | 0.655 |
| Parieto occipital | -0.015 | -0.077 | 0.047 | 0.637 |
| Frontoparietal | -0.021 | -0.083 | 0.042 | 0.508 |
| Salience | -0.010 | -0.073 | 0.052 | 0.744 |
| Cingulo opercular | -0.008 | -0.071 | 0.054 | 0.792 |
| Medial parietal | -0.027 | -0.089 | 0.036 | 0.400 |
| Dorsal attention | -0.022 | -0.085 | 0.041 | 0.489 |
| Ventral attention | -0.016 | -0.079 | 0.047 | 0.614 |
| Visual | -0.004 | -0.066 | 0.059 | 0.906 |
| Somatomotor hand | -0.013 | -0.076 | 0.049 | 0.674 |
| Somatomotor mouth | 0.002 | -0.060 | 0.065 | 0.954 |
| Auditory | -0.007 | -0.070 | 0.056 | 0.820 |
| *p < 0.05  **Bonferroni-corrected p < 0.001 | | | | |

## **Table S9.** Region-based connectivity analysis

| Gordon parcellation region | beta coefficient | lower CI | upper CI | p-value | FDR p-value |
| --- | --- | --- | --- | --- | --- |
| L_None_1 | 0.008 | -0.004 | 0.020 | 0.180 | 0.998 |
| L_None_2 | -0.004 | -0.017 | 0.009 | 0.570 | 0.998 |
| L_None_3 | -0.001 | -0.014 | 0.012 | 0.874 | 0.998 |
| L_None_4 | -0.007 | -0.020 | 0.007 | 0.326 | 0.998 |
| L_None_5 | -0.011 | -0.028 | 0.005 | 0.160 | 0.998 |
| L_None_6 | -0.009 | -0.023 | 0.006 | 0.264 | 0.998 |
| L_None_7 | -0.010 | -0.027 | 0.007 | 0.243 | 0.998 |
| L_None_8 | -0.004 | -0.020 | 0.012 | 0.639 | 0.998 |
| L_None_9 | -0.004 | -0.020 | 0.012 | 0.606 | 0.998 |
| L_None_10 | -0.006 | -0.020 | 0.009 | 0.429 | 0.998 |
| L_None_11 | -0.002 | -0.015 | 0.011 | 0.811 | 0.998 |
| L_None_12 | 0.006 | -0.007 | 0.019 | 0.370 | 0.998 |
| L_None_13 | -0.007 | -0.020 | 0.006 | 0.278 | 0.998 |
| L_None_14 | 0.005 | -0.009 | 0.020 | 0.474 | 0.998 |
| L_None_15 | 0.002 | -0.014 | 0.017 | 0.821 | 0.998 |
| L_None_16 | -0.003 | -0.017 | 0.011 | 0.667 | 0.998 |
| L_None_17 | 0.000 | -0.013 | 0.014 | 0.969 | 0.998 |
| L_None_18 | -0.006 | -0.019 | 0.008 | 0.415 | 0.998 |
| L_None_19 | 0.008 | -0.006 | 0.022 | 0.268 | 0.998 |
| L_None_20 | -0.001 | -0.014 | 0.011 | 0.820 | 0.998 |
| L_None_21 | -0.001 | -0.016 | 0.014 | 0.910 | 0.998 |
| R_None_22 | -0.012 | -0.029 | 0.005 | 0.168 | 0.998 |
| R_None_23 | 0.009 | -0.008 | 0.026 | 0.288 | 0.998 |
| R_None_24 | 0.008 | -0.010 | 0.026 | 0.364 | 0.998 |
| R_None_25 | 0.001 | -0.018 | 0.021 | 0.880 | 0.998 |
| R_None_26 | -0.003 | -0.021 | 0.015 | 0.766 | 0.998 |
| R_None_27 | -0.016 | -0.035 | 0.003 | 0.100 | 0.998 |
| R_None_28 | -0.013 | -0.030 | 0.004 | 0.124 | 0.998 |
| R_None_29 | -0.010 | -0.028 | 0.008 | 0.262 | 0.998 |
| R_None_30 | -0.009 | -0.025 | 0.008 | 0.303 | 0.998 |
| R_None_31 | -0.004 | -0.020 | 0.013 | 0.645 | 0.998 |
| R_None_32 | 0.006 | -0.010 | 0.022 | 0.476 | 0.998 |
| R_None_33 | 0.004 | -0.013 | 0.021 | 0.626 | 0.998 |
| R_None_34 | 0.007 | -0.012 | 0.025 | 0.474 | 0.998 |
| R_None_35 | -0.002 | -0.021 | 0.017 | 0.814 | 0.998 |
| R_None_36 | 0.002 | -0.016 | 0.021 | 0.827 | 0.998 |
| R_None_37 | -0.002 | -0.019 | 0.016 | 0.858 | 0.998 |
| R_None_38 | -0.001 | -0.018 | 0.017 | 0.933 | 0.998 |
| R_None_39 | -0.018 | -0.039 | 0.003 | 0.092 | 0.998 |
| R_None_40 | -0.011 | -0.028 | 0.006 | 0.208 | 0.998 |
| R_None_41 | -0.001 | -0.020 | 0.017 | 0.885 | 0.998 |
| R_None_42 | -0.008 | -0.023 | 0.008 | 0.325 | 0.998 |
| R_None_43 | -0.010 | -0.027 | 0.006 | 0.222 | 0.998 |
| R_None_44 | -0.008 | -0.023 | 0.008 | 0.332 | 0.998 |
| R_None_45 | 0.001 | -0.013 | 0.016 | 0.854 | 0.998 |
| R_None_46 | 0.008 | -0.012 | 0.028 | 0.413 | 0.998 |
| R_None_47 | -0.007 | -0.022 | 0.008 | 0.340 | 0.998 |
| L_Default_1 | 0.003 | -0.016 | 0.021 | 0.782 | 0.998 |
| L_Default_2 | -0.003 | -0.020 | 0.014 | 0.734 | 0.998 |
| L_Default_3 | -0.006 | -0.022 | 0.010 | 0.484 | 0.998 |
| L_Default_4 | 0.006 | -0.012 | 0.024 | 0.499 | 0.998 |
| L_Default_5 | -0.003 | -0.019 | 0.014 | 0.749 | 0.998 |
| L_Default_6 | 0.005 | -0.013 | 0.023 | 0.592 | 0.998 |
| L_Default_7 | 0.000 | -0.018 | 0.019 | 0.969 | 0.998 |
| L_Default_8 | 0.012 | -0.007 | 0.031 | 0.210 | 0.998 |
| L_Default_9 | -0.010 | -0.027 | 0.006 | 0.214 | 0.998 |
| L_Default_10 | 0.003 | -0.015 | 0.020 | 0.747 | 0.998 |
| L_Default_11 | 0.002 | -0.014 | 0.018 | 0.784 | 0.998 |
| L_Default_12 | 0.005 | -0.011 | 0.021 | 0.550 | 0.998 |
| L_Default_13 | 0.000 | -0.017 | 0.017 | 0.993 | 0.998 |
| L_Default_14 | 0.001 | -0.017 | 0.019 | 0.916 | 0.998 |
| L_Default_15 | 0.008 | -0.009 | 0.026 | 0.352 | 0.998 |
| L_Default_16 | 0.003 | -0.015 | 0.021 | 0.727 | 0.998 |
| L_Default_17 | 0.004 | -0.013 | 0.020 | 0.646 | 0.998 |
| L_Default_18 | -0.002 | -0.018 | 0.015 | 0.832 | 0.998 |
| L_Default_19 | 0.002 | -0.015 | 0.018 | 0.854 | 0.998 |
| L_Default_20 | -0.003 | -0.020 | 0.015 | 0.776 | 0.998 |
| R_Default_21 | 0.002 | -0.015 | 0.020 | 0.808 | 0.998 |
| R_Default_22 | -0.009 | -0.026 | 0.008 | 0.308 | 0.998 |
| R_Default_23 | 0.006 | -0.011 | 0.023 | 0.480 | 0.998 |
| R_Default_24 | -0.001 | -0.018 | 0.017 | 0.945 | 0.998 |
| R_Default_25 | 0.001 | -0.016 | 0.018 | 0.926 | 0.998 |
| R_Default_26 | 0.007 | -0.012 | 0.025 | 0.483 | 0.998 |
| R_Default_27 | -0.001 | -0.019 | 0.018 | 0.957 | 0.998 |
| R_Default_28 | -0.007 | -0.022 | 0.008 | 0.377 | 0.998 |
| R_Default_29 | -0.010 | -0.026 | 0.005 | 0.186 | 0.998 |
| R_Default_30 | -0.020 | -0.035 | -0.005 | 0.009* | 0.998 |
| R_Default_31 | 0.002 | -0.015 | 0.019 | 0.827 | 0.998 |
| R_Default_32 | 0.002 | -0.016 | 0.020 | 0.843 | 0.998 |
| R_Default_33 | 0.000 | -0.017 | 0.017 | 0.998 | 0.998 |
| R_Default_34 | -0.002 | -0.019 | 0.014 | 0.768 | 0.998 |
| R_Default_35 | -0.011 | -0.028 | 0.006 | 0.205 | 0.998 |
| R_Default_36 | -0.011 | -0.028 | 0.006 | 0.196 | 0.998 |
| R_Default_37 | -0.008 | -0.025 | 0.009 | 0.377 | 0.998 |
| R_Default_38 | 0.003 | -0.015 | 0.021 | 0.751 | 0.998 |
| R_Default_39 | -0.011 | -0.027 | 0.005 | 0.191 | 0.998 |
| R_Default_40 | -0.015 | -0.031 | 0.001 | 0.063 | 0.998 |
| R_Default_41 | -0.005 | -0.021 | 0.011 | 0.567 | 0.998 |
| L_ParietoOccip_1 | -0.008 | -0.024 | 0.008 | 0.323 | 0.998 |
| L_ParietoOccip_2 | -0.024 | -0.039 | -0.008 | 0.002* | 0.779 |
| L_ParietoOccip_3 | -0.013 | -0.031 | 0.005 | 0.152 | 0.998 |
| L_ParietoOccip_4 | 0.000 | -0.017 | 0.018 | 0.970 | 0.998 |
| R_ParietoOccip_5 | -0.003 | -0.020 | 0.013 | 0.691 | 0.998 |
| R_ParietoOccip_6 | 0.003 | -0.016 | 0.023 | 0.748 | 0.998 |
| R_ParietoOccip_7 | -0.002 | -0.019 | 0.014 | 0.775 | 0.998 |
| R_ParietoOccip_8 | 0.005 | -0.014 | 0.024 | 0.619 | 0.998 |
| L_FrontoParietal_1 | -0.004 | -0.021 | 0.014 | 0.681 | 0.998 |
| L_FrontoParietal_2 | 0.002 | -0.016 | 0.020 | 0.839 | 0.998 |
| L_FrontoParietal_3 | 0.008 | -0.010 | 0.026 | 0.371 | 0.998 |
| L_FrontoParietal_4 | 0.013 | -0.004 | 0.031 | 0.143 | 0.998 |
| L_FrontoParietal_5 | 0.002 | -0.017 | 0.020 | 0.869 | 0.998 |
| L_FrontoParietal_6 | -0.005 | -0.021 | 0.011 | 0.538 | 0.998 |
| L_FrontoParietal_7 | -0.003 | -0.020 | 0.014 | 0.717 | 0.998 |
| L_FrontoParietal_8 | 0.002 | -0.016 | 0.019 | 0.828 | 0.998 |
| L_FrontoParietal_9 | -0.003 | -0.020 | 0.015 | 0.772 | 0.998 |
| R_FrontoParietal_10 | -0.004 | -0.022 | 0.013 | 0.646 | 0.998 |
| R_FrontoParietal_11 | 0.000 | -0.016 | 0.015 | 0.959 | 0.998 |
| R_FrontoParietal_12 | -0.004 | -0.021 | 0.012 | 0.597 | 0.998 |
| R_FrontoParietal_13 | 0.001 | -0.015 | 0.016 | 0.918 | 0.998 |
| R_FrontoParietal_14 | 0.002 | -0.015 | 0.018 | 0.853 | 0.998 |
| R_FrontoParietal_15 | 0.003 | -0.012 | 0.019 | 0.680 | 0.998 |
| R_FrontoParietal_16 | -0.005 | -0.020 | 0.010 | 0.491 | 0.998 |
| R_FrontoParietal_17 | 0.002 | -0.013 | 0.016 | 0.841 | 0.998 |
| R_FrontoParietal_18 | -0.002 | -0.018 | 0.014 | 0.826 | 0.998 |
| R_FrontoParietal_19 | -0.006 | -0.022 | 0.010 | 0.479 | 0.998 |
| R_FrontoParietal_20 | -0.005 | -0.020 | 0.010 | 0.529 | 0.998 |
| R_FrontoParietal_21 | 0.001 | -0.017 | 0.019 | 0.899 | 0.998 |
| R_FrontoParietal_22 | -0.003 | -0.018 | 0.012 | 0.734 | 0.998 |
| R_FrontoParietal_23 | -0.001 | -0.016 | 0.015 | 0.913 | 0.998 |
| R_FrontoParietal_24 | -0.008 | -0.023 | 0.008 | 0.319 | 0.998 |
| L_Salience_1 | 0.001 | -0.016 | 0.018 | 0.931 | 0.998 |
| L_Salience_2 | -0.001 | -0.017 | 0.014 | 0.875 | 0.998 |
| R_Salience_3 | -0.008 | -0.024 | 0.009 | 0.358 | 0.998 |
| R_Salience_4 | -0.012 | -0.027 | 0.003 | 0.122 | 0.998 |
| L_CinguloOperc_1 | -0.009 | -0.026 | 0.007 | 0.279 | 0.998 |
| L_CinguloOperc_2 | 0.009 | -0.007 | 0.026 | 0.275 | 0.998 |
| L_CinguloOperc_4 | 0.000 | -0.017 | 0.017 | 0.973 | 0.998 |
| L_CinguloOperc_5 | 0.003 | -0.014 | 0.020 | 0.735 | 0.998 |
| L_CinguloOperc_6 | 0.005 | -0.013 | 0.022 | 0.603 | 0.998 |
| L_CinguloOperc_7 | 0.004 | -0.014 | 0.022 | 0.664 | 0.998 |
| L_CinguloOperc_8 | -0.003 | -0.020 | 0.015 | 0.779 | 0.998 |
| L_CinguloOperc_9 | 0.011 | -0.009 | 0.030 | 0.275 | 0.998 |
| L_CinguloOperc_10 | 0.010 | -0.009 | 0.028 | 0.307 | 0.998 |
| L_CinguloOperc_11 | -0.005 | -0.021 | 0.012 | 0.576 | 0.998 |
| L_CinguloOperc_12 | -0.004 | -0.021 | 0.013 | 0.633 | 0.998 |
| L_CinguloOperc_13 | 0.006 | -0.011 | 0.022 | 0.486 | 0.998 |
| L_CinguloOperc_14 | 0.007 | -0.010 | 0.023 | 0.441 | 0.998 |
| L_CinguloOperc_15 | 0.007 | -0.010 | 0.024 | 0.414 | 0.998 |
| L_CinguloOperc_16 | 0.012 | -0.005 | 0.030 | 0.154 | 0.998 |
| L_CinguloOperc_17 | 0.001 | -0.015 | 0.017 | 0.877 | 0.998 |
| L_CinguloOperc_18 | -0.006 | -0.022 | 0.010 | 0.459 | 0.998 |
| L_CinguloOperc_19 | 0.005 | -0.012 | 0.023 | 0.542 | 0.998 |
| R_CinguloOperc_21 | -0.001 | -0.017 | 0.014 | 0.878 | 0.998 |
| R_CinguloOperc_22 | 0.004 | -0.012 | 0.020 | 0.637 | 0.998 |
| R_CinguloOperc_24 | -0.009 | -0.026 | 0.008 | 0.312 | 0.998 |
| R_CinguloOperc_26 | 0.003 | -0.013 | 0.020 | 0.688 | 0.998 |
| R_CinguloOperc_27 | -0.010 | -0.026 | 0.007 | 0.254 | 0.998 |
| R_CinguloOperc_28 | 0.001 | -0.017 | 0.019 | 0.936 | 0.998 |
| R_CinguloOperc_29 | 0.006 | -0.012 | 0.024 | 0.491 | 0.998 |
| R_CinguloOperc_30 | 0.010 | -0.008 | 0.028 | 0.260 | 0.998 |
| R_CinguloOperc_31 | -0.004 | -0.021 | 0.014 | 0.684 | 0.998 |
| R_CinguloOperc_32 | -0.013 | -0.030 | 0.003 | 0.114 | 0.998 |
| R_CinguloOperc_33 | 0.007 | -0.010 | 0.025 | 0.419 | 0.998 |
| R_CinguloOperc_34 | 0.004 | -0.014 | 0.023 | 0.650 | 0.998 |
| R_CinguloOperc_35 | 0.004 | -0.012 | 0.020 | 0.642 | 0.998 |
| R_CinguloOperc_36 | 0.003 | -0.014 | 0.021 | 0.714 | 0.998 |
| R_CinguloOperc_37 | -0.004 | -0.022 | 0.013 | 0.611 | 0.998 |
| R_CinguloOperc_38 | 0.000 | -0.017 | 0.018 | 0.962 | 0.998 |
| R_CinguloOperc_39 | -0.001 | -0.018 | 0.015 | 0.871 | 0.998 |
| R_CinguloOperc_40 | -0.001 | -0.014 | 0.012 | 0.924 | 0.998 |
| L_MedialParietal_1 | -0.006 | -0.019 | 0.008 | 0.404 | 0.998 |
| L_MedialParietal_2 | -0.005 | -0.018 | 0.008 | 0.447 | 0.998 |
| L_MedialParietal_3 | 0.010 | -0.002 | 0.021 | 0.112 | 0.998 |
| R_MedialParietal_4 | -0.006 | -0.024 | 0.011 | 0.475 | 0.998 |
| R_MedialParietal_5 | -0.003 | -0.016 | 0.011 | 0.709 | 0.998 |
| L_DorsalAttn_1 | -0.003 | -0.019 | 0.013 | 0.733 | 0.998 |
| L_DorsalAttn_2 | -0.008 | -0.023 | 0.006 | 0.272 | 0.998 |
| L_DorsalAttn_3 | 0.001 | -0.009 | 0.012 | 0.786 | 0.998 |
| L_DorsalAttn_4 | 0.001 | -0.013 | 0.014 | 0.912 | 0.998 |
| L_DorsalAttn_5 | -0.005 | -0.019 | 0.008 | 0.442 | 0.998 |
| L_DorsalAttn_6 | -0.002 | -0.016 | 0.011 | 0.749 | 0.998 |
| L_DorsalAttn_7 | 0.006 | -0.008 | 0.020 | 0.387 | 0.998 |
| L_DorsalAttn_8 | 0.005 | -0.010 | 0.020 | 0.525 | 0.998 |
| L_DorsalAttn_9 | -0.001 | -0.016 | 0.015 | 0.932 | 0.998 |
| L_DorsalAttn_10 | -0.001 | -0.015 | 0.012 | 0.843 | 0.998 |
| L_DorsalAttn_11 | 0.002 | -0.011 | 0.014 | 0.780 | 0.998 |
| L_DorsalAttn_12 | 0.003 | -0.011 | 0.018 | 0.655 | 0.998 |
| L_DorsalAttn_13 | 0.003 | -0.011 | 0.017 | 0.650 | 0.998 |
| L_DorsalAttn_14 | -0.009 | -0.022 | 0.003 | 0.152 | 0.998 |
| L_DorsalAttn_15 | -0.001 | -0.015 | 0.013 | 0.902 | 0.998 |
| L_DorsalAttn_16 | 0.000 | -0.014 | 0.014 | 0.982 | 0.998 |
| L_DorsalAttn_17 | 0.002 | -0.011 | 0.014 | 0.804 | 0.998 |
| L_DorsalAttn_18 | 0.000 | -0.013 | 0.014 | 0.946 | 0.998 |
| L_DorsalAttn_19 | 0.001 | -0.012 | 0.015 | 0.833 | 0.998 |
| R_DorsalAttn_20 | -0.001 | -0.014 | 0.011 | 0.826 | 0.998 |
| R_DorsalAttn_21 | -0.018 | -0.035 | -0.001 | 0.033 | 0.998 |
| R_DorsalAttn_22 | -0.012 | -0.030 | 0.006 | 0.203 | 0.998 |
| R_DorsalAttn_23 | 0.004 | -0.013 | 0.022 | 0.623 | 0.998 |
| R_DorsalAttn_24 | -0.005 | -0.022 | 0.012 | 0.564 | 0.998 |
| R_DorsalAttn_25 | -0.013 | -0.032 | 0.005 | 0.152 | 0.998 |
| R_DorsalAttn_26 | -0.007 | -0.026 | 0.011 | 0.434 | 0.998 |
| R_DorsalAttn_27 | -0.005 | -0.022 | 0.012 | 0.561 | 0.998 |
| R_DorsalAttn_28 | -0.014 | -0.030 | 0.002 | 0.077 | 0.998 |
| R_DorsalAttn_29 | -0.019 | -0.036 | -0.002 | 0.033 | 0.998 |
| R_DorsalAttn_30 | -0.012 | -0.028 | 0.004 | 0.146 | 0.998 |
| R_DorsalAttn_31 | -0.003 | -0.020 | 0.014 | 0.720 | 0.998 |
| R_DorsalAttn_32 | -0.005 | -0.022 | 0.012 | 0.542 | 0.998 |
| L_VentralAttn_1 | -0.025 | -0.046 | -0.004 | 0.020* | 0.998 |
| L_VentralAttn_2 | -0.002 | -0.024 | 0.020 | 0.839 | 0.998 |
| L_VentralAttn_3 | 0.006 | -0.013 | 0.025 | 0.521 | 0.998 |
| L_VentralAttn_4 | -0.005 | -0.024 | 0.014 | 0.610 | 0.998 |
| L_VentralAttn_5 | -0.001 | -0.020 | 0.017 | 0.907 | 0.998 |
| L_VentralAttn_6 | 0.002 | -0.016 | 0.020 | 0.819 | 0.998 |
| L_VentralAttn_7 | 0.000 | -0.019 | 0.019 | 0.993 | 0.998 |
| L_VentralAttn_8 | -0.014 | -0.031 | 0.004 | 0.120 | 0.998 |
| L_VentralAttn_9 | 0.003 | -0.012 | 0.018 | 0.698 | 0.998 |
| L_VentralAttn_10 | -0.015 | -0.031 | 0.000 | 0.057 | 0.998 |
| L_VentralAttn_11 | -0.005 | -0.018 | 0.009 | 0.506 | 0.998 |
| R_VentralAttn_12 | 0.002 | -0.012 | 0.016 | 0.769 | 0.998 |
| R_VentralAttn_13 | 0.004 | -0.009 | 0.018 | 0.531 | 0.998 |
| R_VentralAttn_14 | -0.003 | -0.020 | 0.013 | 0.689 | 0.998 |
| R_VentralAttn_15 | 0.004 | -0.016 | 0.024 | 0.710 | 0.998 |
| R_VentralAttn_16 | -0.003 | -0.020 | 0.013 | 0.681 | 0.998 |
| R_VentralAttn_17 | -0.002 | -0.020 | 0.017 | 0.864 | 0.998 |
| R_VentralAttn_18 | 0.007 | -0.012 | 0.025 | 0.482 | 0.998 |
| R_VentralAttn_19 | 0.004 | -0.013 | 0.021 | 0.646 | 0.998 |
| R_VentralAttn_20 | -0.005 | -0.021 | 0.011 | 0.525 | 0.998 |
| R_VentralAttn_21 | -0.007 | -0.027 | 0.014 | 0.519 | 0.998 |
| R_VentralAttn_22 | -0.013 | -0.031 | 0.004 | 0.139 | 0.998 |
| R_VentralAttn_23 | -0.022 | -0.039 | -0.004 | 0.013 | 0.998 |
| L_Visual_1 | -0.016 | -0.034 | 0.002 | 0.084 | 0.998 |
| L_Visual_2 | -0.006 | -0.024 | 0.013 | 0.541 | 0.998 |
| L_Visual_3 | 0.006 | -0.014 | 0.025 | 0.564 | 0.998 |
| L_Visual_4 | -0.013 | -0.032 | 0.007 | 0.199 | 0.998 |
| L_Visual_5 | -0.008 | -0.027 | 0.011 | 0.394 | 0.998 |
| L_Visual_6 | 0.013 | -0.005 | 0.031 | 0.173 | 0.998 |
| L_Visual_7 | -0.002 | -0.019 | 0.016 | 0.834 | 0.998 |
| L_Visual_8 | 0.007 | -0.011 | 0.024 | 0.453 | 0.998 |
| L_Visual_9 | -0.014 | -0.031 | 0.003 | 0.103 | 0.998 |
| L_Visual_10 | 0.000 | -0.018 | 0.017 | 0.980 | 0.998 |
| L_Visual_11 | -0.002 | -0.018 | 0.015 | 0.851 | 0.998 |
| L_Visual_12 | -0.003 | -0.020 | 0.014 | 0.705 | 0.998 |
| L_Visual_13 | -0.009 | -0.026 | 0.009 | 0.325 | 0.998 |
| L_Visual_14 | -0.001 | -0.020 | 0.017 | 0.908 | 0.998 |
| L_Visual_15 | 0.004 | -0.013 | 0.021 | 0.633 | 0.998 |
| L_Visual_16 | -0.005 | -0.022 | 0.012 | 0.553 | 0.998 |
| L_Visual_17 | -0.006 | -0.022 | 0.010 | 0.457 | 0.998 |
| L_Visual_18 | 0.000 | -0.017 | 0.016 | 0.967 | 0.998 |
| R_Visual_19 | 0.001 | -0.016 | 0.018 | 0.893 | 0.998 |
| R_Visual_20 | 0.000 | -0.016 | 0.017 | 0.975 | 0.998 |
| R_Visual_21 | -0.003 | -0.021 | 0.015 | 0.751 | 0.998 |
| R_Visual_22 | -0.009 | -0.027 | 0.009 | 0.317 | 0.998 |
| R_Visual_23 | -0.002 | -0.018 | 0.015 | 0.852 | 0.998 |
| R_Visual_24 | -0.010 | -0.031 | 0.011 | 0.344 | 0.998 |
| R_Visual_25 | -0.008 | -0.026 | 0.010 | 0.371 | 0.998 |
| R_Visual_26 | -0.002 | -0.016 | 0.013 | 0.826 | 0.998 |
| R_Visual_27 | -0.005 | -0.020 | 0.009 | 0.494 | 0.998 |
| R_Visual_28 | -0.005 | -0.021 | 0.011 | 0.559 | 0.998 |
| R_Visual_29 | -0.004 | -0.021 | 0.013 | 0.660 | 0.998 |
| R_Visual_30 | -0.001 | -0.018 | 0.016 | 0.901 | 0.998 |
| R_Visual_31 | -0.005 | -0.024 | 0.013 | 0.550 | 0.998 |
| R_Visual_32 | -0.012 | -0.030 | 0.005 | 0.165 | 0.998 |
| R_Visual_33 | -0.016 | -0.036 | 0.004 | 0.125 | 0.998 |
| R_Visual_34 | -0.007 | -0.026 | 0.012 | 0.479 | 0.998 |
| R_Visual_35 | -0.005 | -0.021 | 0.011 | 0.548 | 0.998 |
| R_Visual_36 | -0.006 | -0.022 | 0.009 | 0.408 | 0.998 |
| R_Visual_37 | -0.007 | -0.024 | 0.010 | 0.418 | 0.998 |
| R_Visual_38 | -0.001 | -0.019 | 0.017 | 0.890 | 0.998 |
| R_Visual_39 | -0.001 | -0.019 | 0.017 | 0.932 | 0.998 |
| L_SMhand_1 | -0.003 | -0.021 | 0.015 | 0.760 | 0.998 |
| L_SMhand_2 | -0.006 | -0.024 | 0.012 | 0.509 | 0.998 |
| L_SMhand_3 | 0.009 | -0.009 | 0.028 | 0.330 | 0.998 |
| L_SMhand_4 | -0.005 | -0.021 | 0.011 | 0.569 | 0.998 |
| L_SMhand_5 | 0.000 | -0.017 | 0.017 | 0.996 | 0.998 |
| L_SMhand_6 | 0.000 | -0.018 | 0.017 | 0.978 | 0.998 |
| L_SMhand_7 | -0.007 | -0.023 | 0.010 | 0.436 | 0.998 |
| L_SMhand_8 | -0.001 | -0.020 | 0.017 | 0.880 | 0.998 |
| L_SMhand_9 | -0.010 | -0.028 | 0.007 | 0.256 | 0.998 |
| L_SMhand_10 | -0.008 | -0.026 | 0.010 | 0.393 | 0.998 |
| L_SMhand_11 | 0.004 | -0.013 | 0.020 | 0.667 | 0.998 |
| L_SMhand_12 | -0.001 | -0.016 | 0.015 | 0.946 | 0.998 |
| L_SMhand_13 | -0.005 | -0.021 | 0.011 | 0.522 | 0.998 |
| L_SMhand_14 | 0.003 | -0.014 | 0.019 | 0.756 | 0.998 |
| L_SMhand_15 | -0.008 | -0.022 | 0.006 | 0.248 | 0.998 |
| L_SMhand_16 | 0.000 | -0.017 | 0.017 | 0.997 | 0.998 |
| L_SMhand_17 | -0.004 | -0.019 | 0.012 | 0.629 | 0.998 |
| L_SMhand_18 | 0.001 | -0.014 | 0.016 | 0.913 | 0.998 |
| R_SMhand_19 | -0.001 | -0.017 | 0.016 | 0.949 | 0.998 |
| R_SMhand_20 | 0.003 | -0.013 | 0.019 | 0.744 | 0.998 |
| R_SMhand_21 | -0.001 | -0.016 | 0.014 | 0.914 | 0.998 |
| R_SMhand_22 | -0.014 | -0.029 | 0.002 | 0.083 | 0.998 |
| R_SMhand_23 | -0.001 | -0.017 | 0.015 | 0.905 | 0.998 |
| R_SMhand_24 | 0.005 | -0.013 | 0.023 | 0.595 | 0.998 |
| R_SMhand_25 | -0.005 | -0.022 | 0.012 | 0.538 | 0.998 |
| R_SMhand_26 | -0.014 | -0.032 | 0.003 | 0.110 | 0.998 |
| R_SMhand_27 | -0.009 | -0.022 | 0.005 | 0.226 | 0.998 |
| R_SMhand_28 | 0.001 | -0.016 | 0.018 | 0.907 | 0.998 |
| R_SMhand_29 | 0.009 | -0.007 | 0.025 | 0.258 | 0.998 |
| R_SMhand_30 | 0.004 | -0.012 | 0.020 | 0.606 | 0.998 |
| R_SMhand_31 | 0.002 | -0.015 | 0.018 | 0.837 | 0.998 |
| R_SMhand_32 | -0.009 | -0.025 | 0.007 | 0.262 | 0.998 |
| R_SMhand_33 | 0.010 | -0.006 | 0.026 | 0.212 | 0.998 |
| R_SMhand_34 | -0.002 | -0.017 | 0.014 | 0.846 | 0.998 |
| R_SMhand_35 | 0.004 | -0.013 | 0.020 | 0.652 | 0.998 |
| R_SMhand_36 | 0.003 | -0.015 | 0.021 | 0.724 | 0.998 |
| R_SMhand_37 | 0.004 | -0.015 | 0.024 | 0.659 | 0.998 |
| R_SMhand_38 | -0.010 | -0.028 | 0.008 | 0.259 | 0.998 |
| L_SMmouth_1 | 0.000 | -0.018 | 0.018 | 0.984 | 0.998 |
| L_SMmouth_2 | 0.002 | -0.016 | 0.020 | 0.848 | 0.998 |
| L_SMmouth_3 | -0.003 | -0.020 | 0.014 | 0.720 | 0.998 |
| L_SMmouth_4 | 0.003 | -0.014 | 0.020 | 0.711 | 0.998 |
| R_SMmouth_5 | 0.006 | -0.012 | 0.023 | 0.518 | 0.998 |
| R_SMmouth_6 | 0.010 | -0.007 | 0.026 | 0.244 | 0.998 |
| R_SMmouth_7 | 0.005 | -0.011 | 0.022 | 0.546 | 0.998 |
| R_SMmouth_8 | 0.011 | -0.007 | 0.029 | 0.216 | 0.998 |
| L_Auditory_1 | 0.002 | -0.014 | 0.019 | 0.781 | 0.998 |
| L_Auditory_2 | 0.006 | -0.012 | 0.023 | 0.528 | 0.998 |
| L_Auditory_3 | -0.001 | -0.018 | 0.016 | 0.873 | 0.998 |
| L_Auditory_4 | 0.008 | -0.010 | 0.026 | 0.380 | 0.998 |
| L_Auditory_5 | 0.005 | -0.012 | 0.021 | 0.569 | 0.998 |
| L_Auditory_6 | -0.002 | -0.019 | 0.015 | 0.815 | 0.998 |
| L_Auditory_7 | -0.006 | -0.021 | 0.010 | 0.489 | 0.998 |
| L_Auditory_8 | 0.000 | -0.016 | 0.016 | 0.971 | 0.998 |
| L_Auditory_9 | 0.005 | -0.011 | 0.022 | 0.509 | 0.998 |
| L_Auditory_10 | -0.008 | -0.025 | 0.010 | 0.395 | 0.998 |
| L_Auditory_11 | 0.006 | -0.010 | 0.021 | 0.487 | 0.998 |
| L_Auditory_12 | -0.007 | -0.023 | 0.010 | 0.446 | 0.998 |
| R_Auditory_13 | 0.001 | -0.017 | 0.019 | 0.894 | 0.998 |
| R_Auditory_14 | 0.011 | -0.007 | 0.030 | 0.223 | 0.998 |
| R_Auditory_15 | 0.000 | -0.016 | 0.017 | 0.954 | 0.998 |
| R_Auditory_16 | -0.001 | -0.018 | 0.017 | 0.921 | 0.998 |
| R_Auditory_17 | -0.006 | -0.022 | 0.010 | 0.452 | 0.998 |
| R_Auditory_18 | -0.002 | -0.020 | 0.015 | 0.794 | 0.998 |
| R_Auditory_19 | -0.001 | -0.018 | 0.016 | 0.938 | 0.998 |
| R_Auditory_20 | -0.002 | -0.017 | 0.014 | 0.847 | 0.998 |
| R_Auditory_21 | 0.006 | -0.010 | 0.022 | 0.470 | 0.998 |
| R_Auditory_22 | 0.000 | -0.018 | 0.017 | 0.962 | 0.998 |
| R_Auditory_23 | 0.002 | -0.015 | 0.019 | 0.810 | 0.998 |
| R_Auditory_24 | -0.003 | -0.020 | 0.013 | 0.687 | 0.998 |
| *Indicates p<0.05  **Indicates p-fdr corrected p<0.05 | | | | | |

# SUPPLEMENTARY FIGURES

**
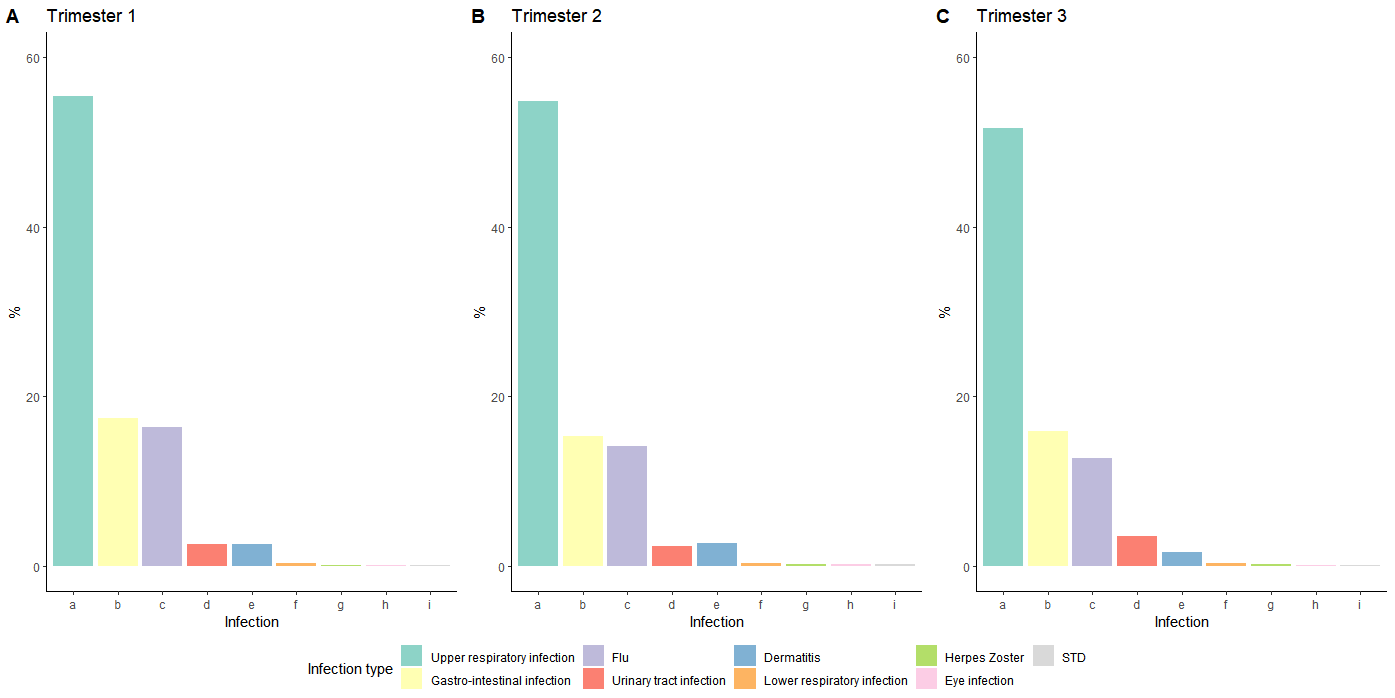
**

## **Figure S1.** Distribution different infection types. Figures 1A-C show the distribution of the infection types for trimester 1, 2 and 3, respectively.


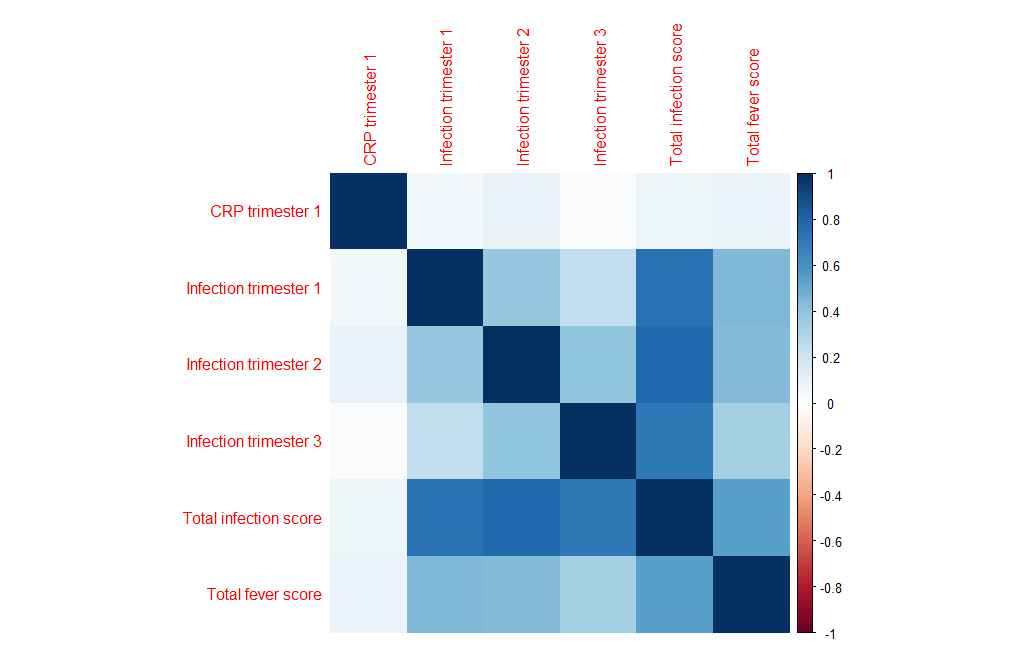


## **Figure S2.** Correlation between C-reactive protein levels <18 weeks of gestation (1 fixed time point measurement available in Generation R) and total and trimester-based prenatal infection scores.


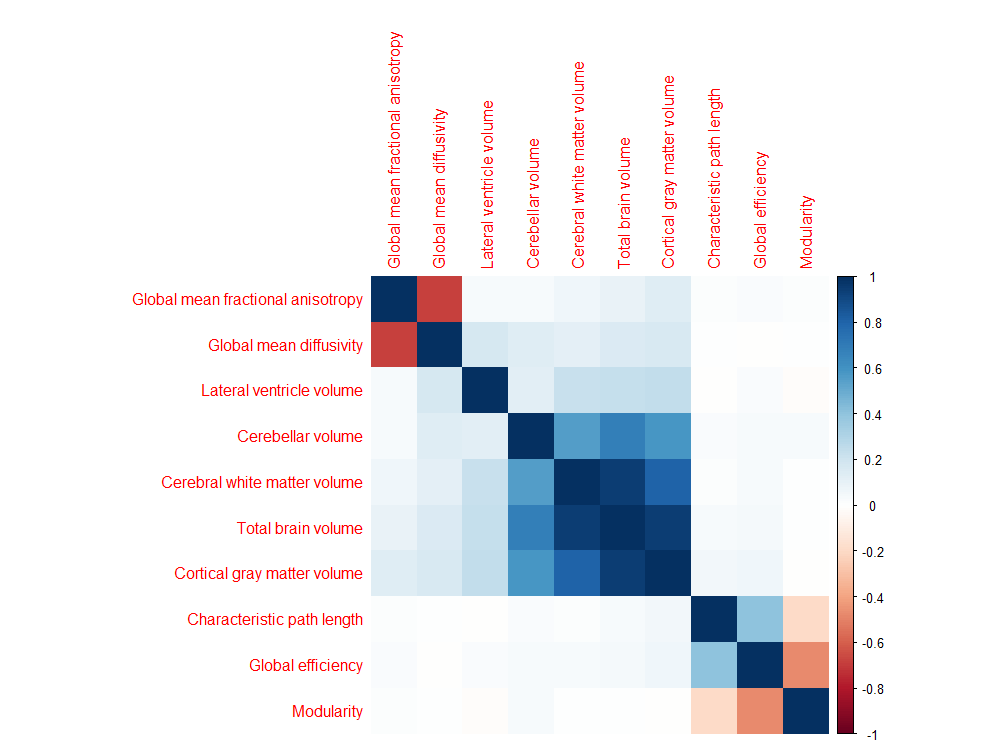


## **Figure S3**. Correlation plot of global brain outcomes.

## **Figure S4**. Correlation plot of focal brain outcomes.
